# Passive acoustic listening stations (PALS) show rapid onset of ecological effects of harmful algal blooms in real time

**DOI:** 10.1038/s41598-020-74647-z

**Published:** 2020-10-20

**Authors:** Athena M. Rycyk, Reny B. Tyson Moore, Randall S. Wells, Katherine A. McHugh, Elizabeth J. Berens McCabe, David A. Mann

**Affiliations:** 1 Division of Natural Sciences, New College of Florida, Sarasota, FL 34243 USA; 2 Chicago Zoological Society’s Sarasota Dolphin Research Program, c/o Mote Marine Laboratory, Sarasota, FL 34236 USA; 3 Loggerhead Instruments, Inc, Sarasota, FL 34238 USA

**Keywords:** Ecology, Marine biology

## Abstract

Monitoring ecological changes in marine ecosystems is expensive and time-consuming. Passive acoustic methods provide continuous monitoring of soniferous species, are relatively inexpensive, and can be integrated into a larger network to provide enhanced spatial and temporal coverage of ecological events. We demonstrate how these methods can be used to detect changes in fish populations in response to a *Karenia brevis* red tide harmful algal bloom by examining sound spectrum levels recorded by two land-based passive acoustic listening stations (PALS) deployed in Sarasota Bay, Florida, before and during a red tide event. Significant and temporally persistent decreases in sound spectrum levels were recorded in real time at both PALS in four frequency bands spanning 0.172–20 kHz after *K. brevis* cells were opportunistically sampled near the stations. The decrease in sound spectrum levels and increase in *K. brevis* cell concentrations also coincided with decreased catch per unit effort (CPUE) and species density per unit effort (SDPUE) data for non-clupeid fish and soniferous fish species, as well as increased reports of marine mammal mortalities in the region. These findings demonstrate how PALS can detect and report in real time ecological changes from episodic disturbances, such as harmful algal blooms.

## Introduction

*Karenia brevis* is a marine dinoflagellate that causes red tide, a type of harmful algal bloom (HAB), in the Gulf of Mexico^1^. *K. brevis* produces a suite of neurotoxin compounds called brevetoxins that can be inhaled or transferred through the food web, sometimes resulting in massive fish kills and mortality of marine megafauna, such as Florida manatees (*Trichechus manatus latirostris*), sea turtles (*Lepidochelys kempii*, *Caretta caretta*, and *Chelonia mydas*), seabirds, and common bottlenose dolphins (*Tursiops truncatus*)^2–5^. Impacts of HAB events can extend beyond marine life and affect human health and livelihoods. Reduction in the fish population, the smell of dead marine organisms, and the negative health impacts of aerosolized brevetoxins on humans can reduce commercial- and recreational-human use of the marine environment and coastlines during these events^6–8^.

Globally, there has been an apparent increase in HAB occurrence in recent decades, coupled with increasing frequency, intensity, duration, and geographic distribution of HAB impacts within affected areas^9,10^. Potential causes of this apparent increase include a number of factors, including increased scientific awareness and observer effort (including improved detection technology), and human activities contributing to nutrient loading, eutrophication, and global climate change, which may affect bloom dynamics^9,10^. *K. brevis* bloom dynamics seem to be particularly complex; blooms appear with near annual frequency along the West Florida Shelf and are supported by a wide range of nutrient sources, with no clear source or other environmental factor serving as a primary contributor to extended bloom durations^11,12^. Therefore *K. brevis* bloom dynamics present a recurring threat to coastal ecosystems and communities, making it prudent to explore effective and inexpensive tools to detect and monitor these events. While satellite imagery and remote sensing techniques can be used to monitor the presence and movement of HABs^13^, these tools are not capable of providing fine-scale information near-shore in estuarine habitats or detecting the ecological effects that such events may have on a system.

Current methods for monitoring the biological and ecological impacts of HABs and other environmental disturbances can be resource- and time-consuming. The status of fish populations can be examined through population surveys, which commonly involve using seine or trawl nets to capture and document fish abundance at selected sites^5,14^, and/or through tagging studies, which can be used to monitor movements of individuals over time. Changes in marine mammal population structure and movement patterns can be assessed via boat-based photo-identification or aerial surveys and/or by tracking tagged individuals^15–19^. Changes in dolphin health and body condition can be evaluated through capture-release health assessments^20^. Mortality of species can be assessed through carcass salvage and necropsy programs^21,22^. While these methods to track impacts related to changes in biological and anthropogenic changes in coastal bays and estuaries are effective at detecting population changes in marine megafauna and fish, they are limited in their temporal and spatial resolution to when and where surveys and programs are conducted, and they cannot provide continuous, fine-scale data on changes in ecological patterns, such as changes in individual habitat use, distributions, and trophic interactions.

Here we demonstrate the use of Passive Acoustic Monitoring (PAM) methods to track biological and anthropogenic changes resulting from HABs, in coastal bays, sounds, and estuaries. PAM is a powerful tool that can be used to detect and monitor biological and anthropogenic activities in aquatic environments^23^. Many species including soniferous (sound-producing) species of invertebrates, fish, and mammals use sound for social interactions, foraging, navigation, threat avoidance, and mating. Consequently, detection of biological sound with PAM can provide information about species presence, distribution, behavior, and, in some cases, demography (e.g., size, sexual maturity)^24,25^. Acoustic detections can be used to investigate the biology of a single species or, when taken together, reflect biological activity across species and trophic levels. For example, the diversity of sounds produced in an aquatic environment can reflect biodiversity^26–28^. Levels of human activity can also be detected through recordings of anthropogenic noise (e.g., boat noise)^29^.

**Figure 1:**
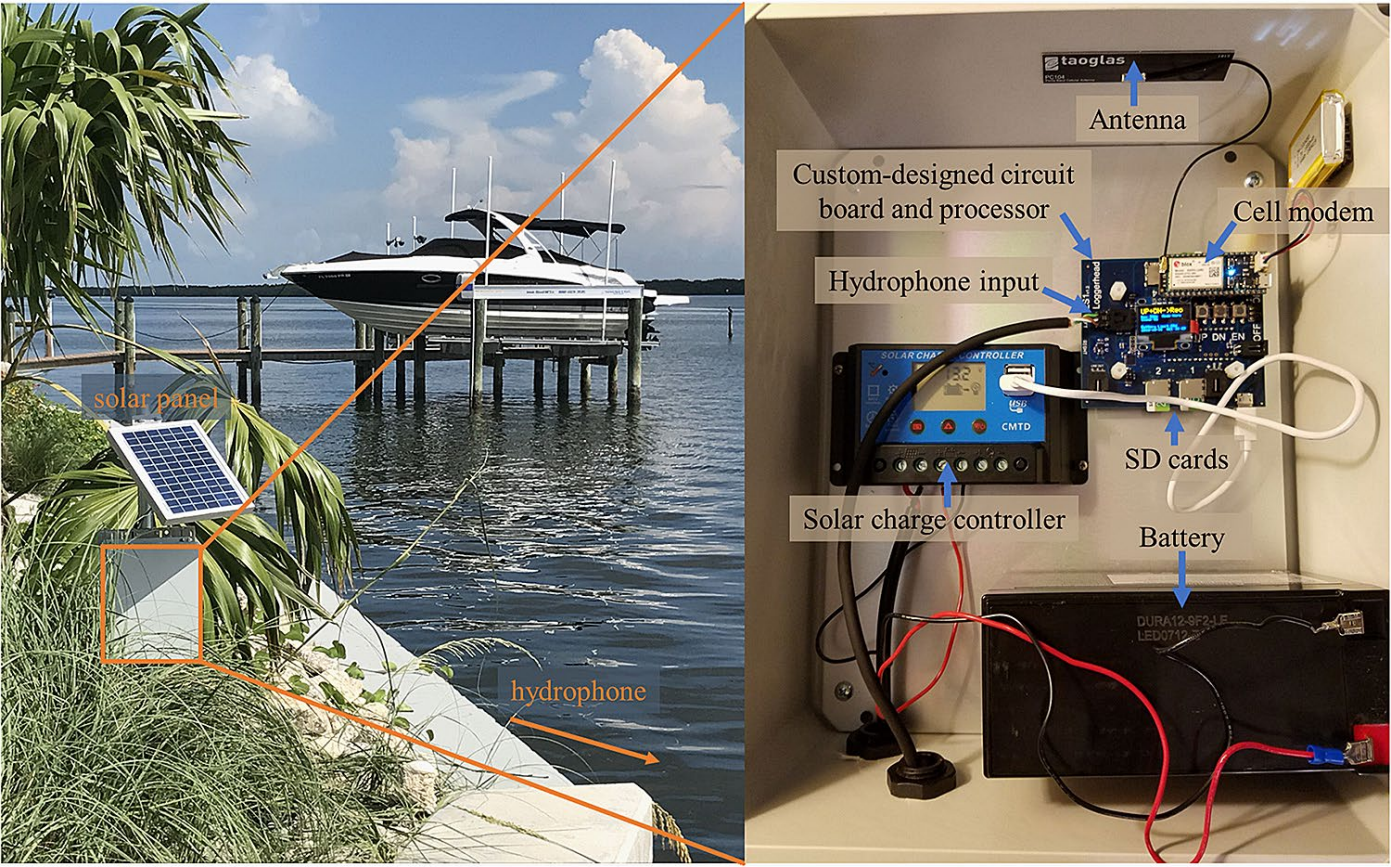
The first Passive Acoustic Listening Station (PALS) on Longboat Key (LBK), Florida. On the left, the station equipment is boxed in orange. The solar panel charges the battery inside the grey box that houses the recording equipment (pictured on the right). The hydrophone is deployed in the water (orange arrow) and a cable runs over the seawall connecting the grey box and hydrophone.

Here, we demonstrate the use of an automated PAM system to detect and monitor ecological effects of HABs through a case study whereby two land-based passive acoustic listening stations (PALS, Loggerhead Instruments, Inc.) deployed near Sarasota Bay, Florida (Fig. 1) recorded biological and anthropogenic sound before and during a severe HAB that occurred in the region in 2018. Our results demonstrate that PAM can be a valuable tool for monitoring the response of ecosystems to environmental perturbations, such as those caused by HABs, and should be considered for implementation in regions susceptible to environmental disturbance.

## Results

### Karenia brevis cell concentrations

**Figure 2:**
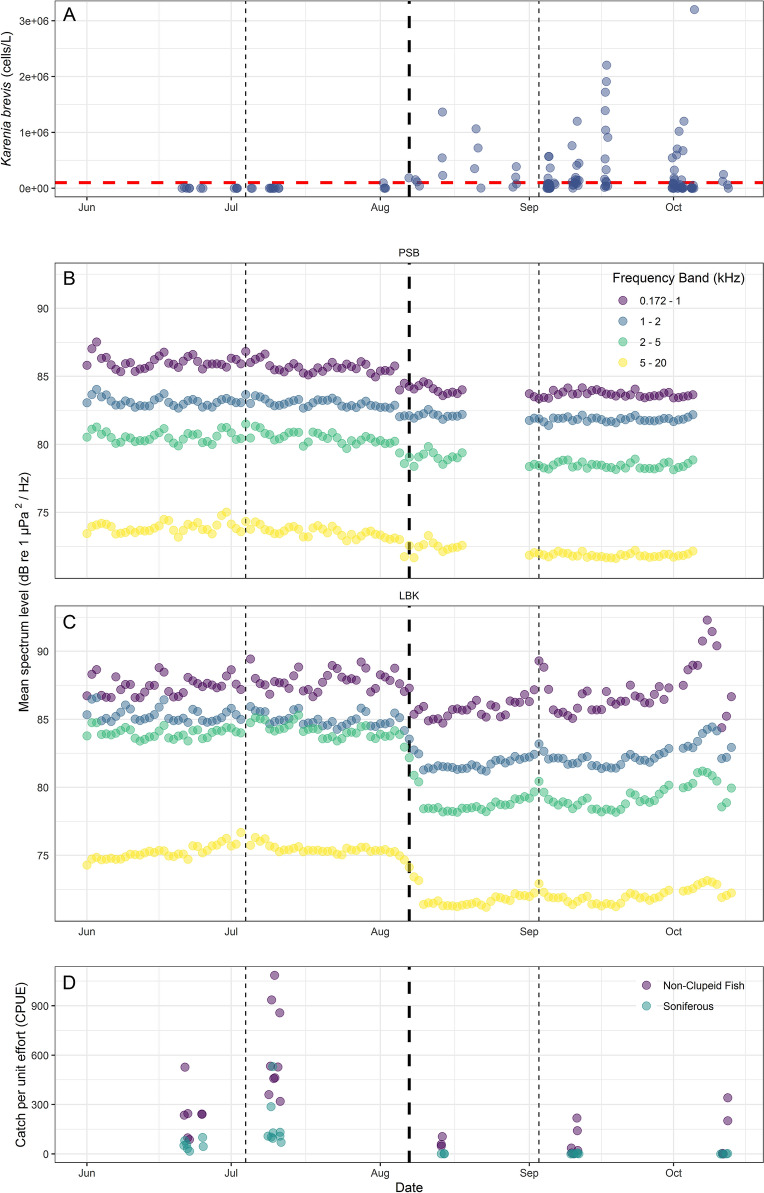
The number of *Karenia brevis* cells/L (**A**), daily mean sound spectrum levels (dB re 1 μPa^2^/Hz) at the PSB PALS (**B**) and LBK PALS (**C**), and non-clupeid fish and soniferous fish CPUEs within the study limits between 1 June 2018 and 13 October 2018. The red horizontal dashed line in panel A represents 100,000 *Karenia brevis* cells/L, the threshold typically considered to represent fish kills^5,30,36^. The thin dashed vertical lines in all panels correspond to holidays that occurred during the study period (4 July 2018—USA Independence Day and 3 September 2018–USA Labor Day, respectively), which may contribute to higher levels of anthropogenic noise recorded on these days. Other peaks in average sound spectrum levels, particularly during the day, generally correspond with weekends. Increases in SPLs observed at the LBK PALS in October were a result of physical noise (i.e., increased wave action) rather than biological noise. The thick dashed vertical line in all panels corresponds to 7 August 2018; the first day that *Karenia brevis* cell counts were measured to be ≥ 100,000/L. Note: one *Karenia brevis* cell count (13,760,000 #/L recorded on 02 October 2018) was excluded from panel A as it was much larger than all other records and skewed the visualization of the data.

**Figure 3:**
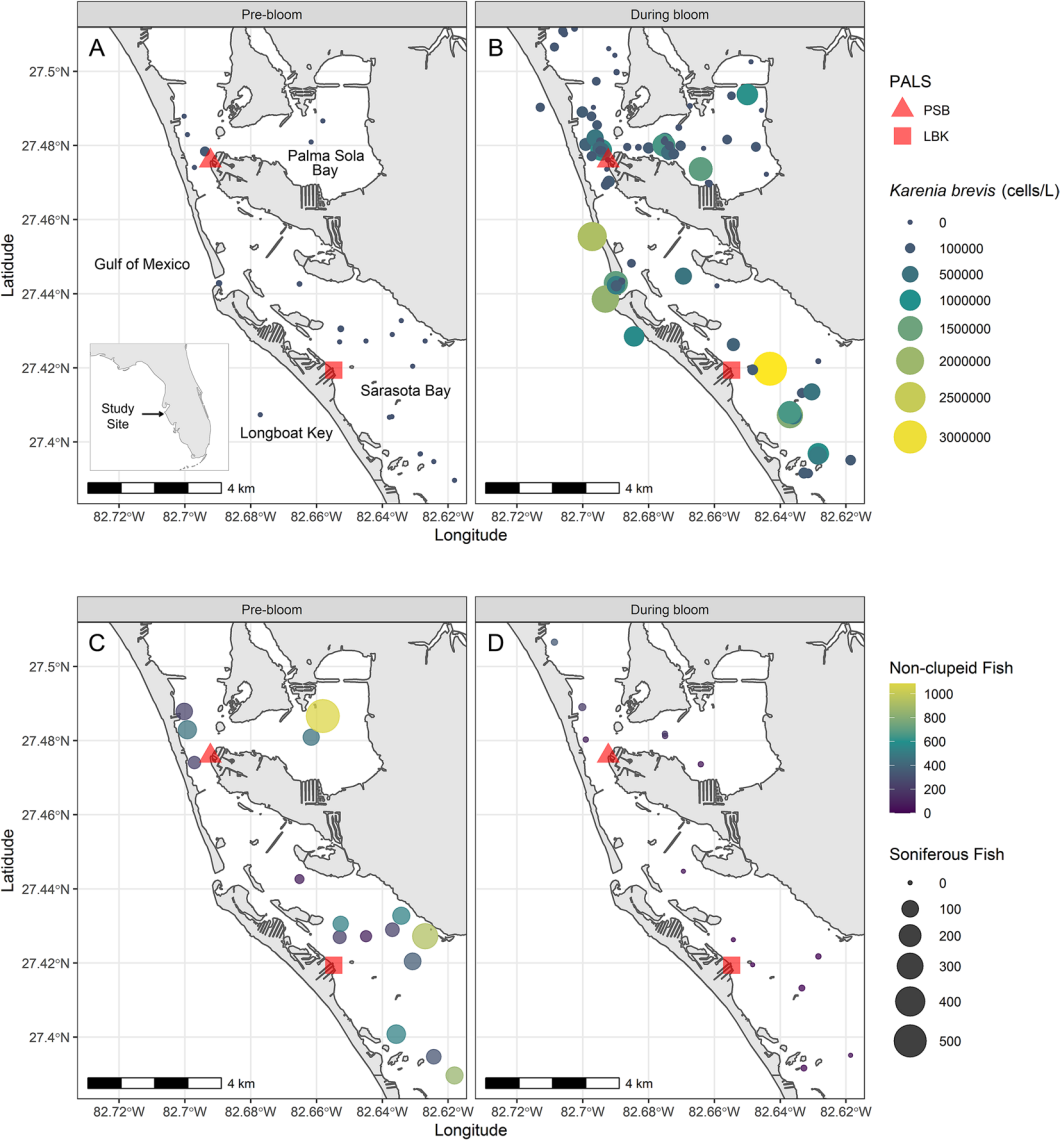
Locations of acoustic and red tide monitoring stations within the study site, along with the *Karenia brevis* cell counts (**A**,**B**) and non-clupeid and soniferous fish catch per unit effort (**C**,**D**) pre- and during bloom. Maps were made in R v3.5.2 using the following packages: *cowplot*^55^, *rnaturalearth*^57^, *sf*^58^, *tidyverse*^59^, and *viridis*^60^ and the Florida Shoreline (1 to 40,000 Scale) shapefile available from the Florida Fish and Wildlife Conservation Commission GIS & Mapping Data Downloads (https://geodata.myfwc.com/)^61^. Note: one *Karenia brevis* cell count (13,760,000 #/L recorded during the bloom at 27.42392 and − 82.65965) was excluded from panel B as it was much larger than all other records and skewed the visualization of the data.

As part of ongoing red-tide monitoring studies in the Sarasota Bay region, 136 water samples were collected between 16 June 2018 and 12 October 2018 within our study area in and near Sarasota Bay, Florida. These water samples were used to measure *K. brevis* cell count concentrations (Figs. 2A and 3A,B). While some *K. brevis* cells were detected as early as 21 June 2018 (Figs. 2A and 3A,B), the first day that cells were recorded to be ≥ 100,000 cells/L (i.e., the threshold for fish kill levels^5,14,30–32^; hereafter referred to as ichthyotoxic levels) in our study region was 7 August 2018 (186,000 cells/L; Figs. 2A and 3A,B). During the study, cell counts ranged from 0–13,760,000 cells/L (mean [median] = 338,213 [60,000] cells/L), with cell counts being significantly greater after 7 August 2018 (hereafter referred to as during bloom: mean [median, range] = 413,351 [82,000, 0–13,760,000] cells/L (*n* = 111)) than before (hereafter referred to as pre-bloom: 4600 [0, 0–96,000] cells/L (*n* = 25)) (Wilcoxon rank sum test: *W* = 320.5, *P* = 1.3 × 10^–9^).

### Acoustic recordings

**Figure 4:**
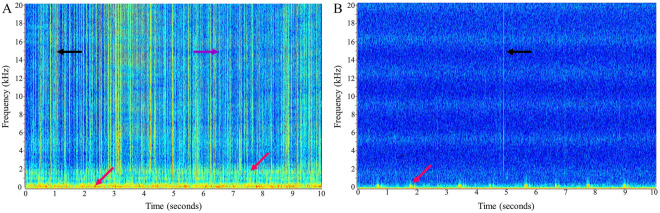
Representative spectrograms of the soundscape at the PSB station pre-bloom (**A**, 1 August 2018) and during bloom (**B**, 1 September 2018). The black arrows point to snapping shrimp snaps (broadband [vertical lines] clicks of varying intensity and interval), the purple arrow points to common bottlenose dolphin echolocation (broadband [narrower vertical lines] clicks of similar intensity and interval), and red arrows point out sounds produced by fish. There are more biological sounds in the pre-bloom spectrogram than the during bloom spectrogram.

PALS located at the entrance to Palma Sola Bay (PSB) and the bay side of Longboat Key (LBK) recorded near continuous audio for 114 and 132 days, respectively, between 1 June 2018 and 13 October 2018 (Figs. 2 and 3). Gaps in data records occurred primarily due to power limitations with the solar panels or issues with the circuit board restarting, which have since been resolved. Mean sound spectrum levels (dB re 1 μPa^2^/Hz) were calculated across every 5-min interval continuously for four distinct frequency bands (0.172–1 kHz, 1–2 kHz, 2–5 kHz, and 5–20 kHz) and were reported in real time over a cell phone network to a cloud database and website (Figs. 2B,C and 4). The number of data records received per day were 276 [288, 67–289] (*n* = 36,895 records and *n* = 114 days) and 280 [288, 6–289] (*n* = 31,484 records and *n* = 132 days) at PSB and LBK, respectively. Mean sound spectrum levels were variable at each PALS and for each frequency band, with the lowest frequency band (0.172–1 kHz) having the highest mean spectrum levels, and the highest frequency band (5–20 kHz) having the lowest mean spectrum levels (Table 1, Fig. 2B,C). All bands were significantly louder during the day than at night at both stations (Table 1).

**Table 1:** Overall (PSB: *n* = 31,484; LBK: *n* = 36,895), daytime (PSB: *n* = 16,563; LBK: *n* = 19,458), and nighttime (PSB: *n* = 14,951; LBK: *n* = 17,437) mean [median, range] values of the five-minute mean sound spectrum levels (dB re 1 μPa^2^/Hz) reported for each frequency band recorded at the PSB and LBK Passive Acoustic Listening Stations (PALS) during the study.

PALS	Frequency band (kHz)	All	Day	Night	Day versus night
		Mean [median, range] dB re 1 μPa^2^/Hz			Wilcoxon rank sum test
PSB	0.172–1	85.1 [85, 83–104]	85.5 [85, 83–104]	84.7 [84, 83–103]	*W* = 163,845,512, *P* = 2.2 × 10^–16^
	1–2	82.6 [82, 81–95]	82.8 [83, 81–95]	82.4 [82, 81–88]	*W* = 142,125,415, *P* = 2.2 × 10^–16^
	2–5	79.8 [80, 78–89]	80.0 [80, 78–89]	79.5 [80, 78–86]	*W* = 139,021,214, *P* = 2.2 × 10^–16^
	5–20	73.1 [73, 71–83]	73.1 [73, 71–82]	73.1 [73, 71–83]	*W* = 115,144,463, *P* = 2.2 × 10^–16^
LBK	0.172–1	87.2 [86, 84–105]	87.8 [87, 84–105]	86.6 [86, 84–105]	*W* = 224,150,390, *P* = 2.2 × 10^–16^
	1–2	83.8 [84, 81–94]	84.1 [84, 81–94]	83.5 [84, 81–90]	*W* = 188,959,069, *P* = 2.2 × 10^–16^
	2–5	82.0 [82, 78–90]	82.0 [82, 78–90]	81.9 [82, 78–88]	*W* = 171,651,501, *P* = 0.047
	5–20	73.8 [74, 71–81]	73.6 [74, 71–79]	74.0 [74, 71–81]	*W* = 156,805,375, *P* = 2.2 × 10^–16^

Results of Wilcoxon rank sum tests comparing daytime and nighttime mean sound spectrum levels are also included.

Mean sound spectrum levels were significantly different pre- and during bloom for each frequency band at each PALS (PSB and LBK) (Table 2, Figs. 2B,C and 4). At PSB, mean spectrum levels decreased on average 2.2 dB re 1 μPa^2^/Hz in the 0.172–1 kHz band, 1.2 dB re 1 μPa^2^/Hz in the 1–2 kHz band, 1.9 dB re 1 μPa^2^/Hz in the 2–5 kHz band, and 1.7 dB re 1 μPa^2^/Hz in the 5–20 kHz band after 7 August 2018 (Table 2). At LBK these decreases were greater for most bands with mean spectrum level decreases equal to 0.9 dB re 1 μPa^2^/Hz in the 0.172–1 kHz band, 3.0 dB re 1 μPa^2^/Hz in the 1–2 kHz band, 4.9 dB re 1 μPa^2^/Hz in the 2–5 kHz band, and 3.3 dB re 1 μPa^2^/Hz in the 5–20 kHz band (Table 2). Increases in SPLs for all four bands observed in October at LBK were verified by listening to the station’s raw recordings to be a result of physical noise during low tides (i.e., increased wave action likely related to an offshore hurricane’s influence on wind speed and direction) rather than biological noise. Similar increases were not readily observed at PSB because the PALS stopped recording on 5 October 2020, generally before the increased wave action occurred.

**Table 2:** Mean [median, range] sound spectrum levels (dB re 1 μPa^2^/Hz) pre- (PSB: *n* = 12,825; LBK; *n* = 18,515) and during (PSB: *n* = 18,659; LBK: *n* = 18,380) the *K. brevis* bloom in the study region recorded at the PSB and LBK Passive Acoustic Listening Stations (PALS), as well as results from corresponding Wilcoxon rank sum tests.

PALS	Frequency band (kHz)	Pre-bloom	During bloom	Wilcoxon rank sum test
		Mean [median, range] dB re 1 μPa^2^/Hz		
PSB	0.172–1	85.9 [86, 83–104]	83.7 [83, 83–94]	*W* = 210,960,791, *P* = 2.2 × 10^–16^
	1–2	83.1 [83, 82–95]	81.9 [82, 81–90]	*W* = 206,869,113, *P* = 2.2 × 10^–16^
	2–5	80.5 [80, 78–89]	78.6 [78, 78–87]	*W* = 220,067,517, *P* = 2.2 × 10^–16^
	5–20	73.7 [74, 71–83]	72.0 [72, 71–81]	*W* = 215,530,631, *P* = 2.2 × 10^–16^
LBK	0.172–1	87.7 [87, 84–105]	86.8 [86, 84–100]	*W* = 230,963,972, *P* = 2.2 × 10^–16^
	1–2	85.2 [85, 83–94]	82.2 [82, 81–91]	*W* = 328,398,292, *P* = 2.2 × 10^–16^
	2–5	84.1 [84, 80–90]	79.2 [79, 78–90]	*W* = 336,227,230,* P* = 2.2 × 10^–16^
	5–20	75.3 [75, 73–81]	72.0 [72, 71–78]	*W* = 336,820,248, *P* = 2.2 × 10^–16^

All values recorded before 7 August 2018 were considered pre-bloom, while all values recorded on or after this day were considered during bloom.

### Fish and environmental samples

Fish in the study region were sampled as part of ongoing long-term seasonal surveys of dolphin prey fish availability^5^, with extended extra-seasonal effort to investigate the 2018 HAB. Catch per unit effort (CPUE) and species density per unit effort (SDPUE) data for non-clupeid fish and soniferous fish species (Table 3) were available from twenty-nine purse-seine deployments deployed in the study region during our study period (Figs. 2D, and 3C,D). There was a total of 9281 non-clupeid and 1829 soniferous fish caught during the study period across sampling sites. Mean [median, range] CPUE’s during the study were 289.5 [235, 0–1085] for non-clupeid fish, and 67.7 [32, 0–530] for soniferous fish. There was a total of 53 non-clupeid and 10 soniferous fish species caught during the study period across sampling sites. Non-clupeid and soniferous SDPUE were 10.3 [11, 0–22] and 2.9 [3, 0–9], respectively. CPUEs and SDPUEs for both non-clupeid fish and soniferous fish were significantly different pre- (*n* = 16) and during (*n* = 13) the *K. brevis* bloom. Non-clupeid fish CPUE and SDPUE pre-bloom were 451.0 [410, 87–1085] and 14.6 [14, 9–22], respectively; while during the bloom CPUE and SDPUE were 90.8 [48, 0–341] and 5.0 [4, 0–12], respectively (*W* = 199, *P* = 3.2 × 10^–5^). Soniferous fish CPUE and SDPUE pre-bloom were 121.6 [98, 16–530] and 4.4 [4, 2–9], respectively, while during the bloom were 1.4 [1.0, 0–6] and 1.0 [1, 0–3], respectively (*W* = 203.5, *P* = 1.1 × 10^–5^).

**Table 3:** Fish species classified as soniferous in the fish surveys.

Soniferous fish species
Crevalle jack, *Caranx hippos*
Gulf toadfish, *Opsanus beta*
Hardhead catfish, *Arius felis*
Leopard searobin, *Prionotus scitulus*
Pigfish, *Orthopristis chrysoptera*
Red drum, *Sciaenops ocellatus*
Silver perch, *Bairdiella chrysoura*
Spot, *Leiostomus xanthurus*
Spotted seatrout, *Cynoscion nebulosus*
White grunt, *Haemulidae plumieri*

Bottom temperature, dissolved oxygen content (DO), and salinity were measured at all twenty-nine fish sampling sites with samples being taken from a mean [median, range] depth of 1.3 [1.2, 0.8–2.1] m. Bottom temperature and salinity were significantly different during the pre-bloom period (*n* = 15) and during the *K. brevis* bloom (*n* = 13) (*W* = 147.5, *P* = 0.02 and *W* = 185.5, *P* = 5.5 × 10^–5^, respectively) with pre-bloom measurements of 30.7 [30.8, 29.6–31.8] °C and 32.1 [32.3, 29.7–33.4] PPT, respectively; and bloom measurements of 29.9 [29.7, 27.8–33.0] °C and 29.3 [29.1, 27.7–32] PPT, respectively. Bottom DO was statistically similar between both periods: 4.5 [5.3, 0.52–10.1] (pre-bloom) and 5.8 [5.6, 2.8–8.8] (during bloom) (*W* = 73, *P* = 0.27).

## Discussion

*K. brevis* blooms are unpredictable phenomena that can cause drastic ecological changes and can negatively affect the health and survival of marine species, as well as impacting both economical- and recreational-use of marine ecosystems by humans. Inexpensive and continuous methods of monitoring ecological changes associated with *K. brevis* blooms are needed. Our passive acoustic monitoring of the local soundscape at two of our initial PALS sites included recordings before and during a *K. brevis* HAB event. We compared mean received sound spectrum levels to *K. brevis* concentrations and environmental data, as well as non-clupeid and soniferous fish CPUEs and SDPUEs from ongoing, long-term fish surveys conducted in the vicinity during the same time period. A decrease in mean sound spectrum levels across frequency bands coincided with the presence and increase in *K. brevis* concentrations and decrease in non-clupeid and soniferous fish CPUE and SDPUE (Fig. 2). The mean spectrum level decrease occurred across all examined frequency bands suddenly as *K. brevis* concentrations increased. The decrease in mean spectrum level occurred for both day and night suggesting the decrease was not just a reduction in boat use (concentrated during the day) during the bloom.

A reduction in biological sound production can indicate mortality of the sound-producing species, a shift in species distribution, or a change in sound production patterns. First, we consider mortality of fish during this period. Florida Fish and Wildlife Conservation Commission maintains a database of publicly reported fish kills^33^. While these reports do not specify soniferous fish, reports categorized as fish kills with a probable cause of red tide in Manatee County, where the PALS sites were located, increased from 34 before *K. brevis* concentrations reached ichthyotoxic levels (i.e., 100,000 cells/L) (1 June 2018–6 August 2018) to 63 during the bloom period examined in this study (7 August 2018–13 October 2018). Prior to 7 August 2018 a *K. brevis* count of 96,000 cells/L was measured in the study area on 2 August 2018 and 33 of the 34 fish kill reports before 7 August 2018 occurred after 2 August 2018. These fish kills could be red tide related as Gannon et al. (2009) found changes in abundance and fish species density below the 100,000 cells/L threshold during a previous HAB in the region. Fish kill reports are anecdotal in nature, but can help interpret data collected in a standardized, scientific manner. Findings from standardized fish surveys in the current study found a drastic decrease in CPUE and SDPUE for all non-clupeid and soniferous fish species during the same period. The decrease in mean CPUE for soniferous fish was particularly striking (121.6 pre-bloom to 1.4 during bloom) and suggests widespread mortality of sound producing fish in this region.

Second, the decrease in sound production observed may be a result of sublethal impacts on soniferous species. Organisms may shift their distribution to areas with lower concentrations of *K. brevis* and/or higher concentrations of prey, and/or organisms may change their behavior in a manner that reduces sound production. The red tide observed in this study started in the southern portion of the study area and moved north, resulting in a patchy spatial extent that covered the immediate study area, but little evidence that it extended much farther north than the very southern portions of Tampa Bay. If emigration occurred, fish would have had to detect and avoid patches of *K. brevis* or brevetoxin and move tens of kilometers away to avoid the bloom. It is unknown whether or not fish can detect and avoid *K. brevis* or its associated brevetoxins. Conversely, it is well known that brevetoxins cause mortality in fish^34,35^ (reviewed by Landsberg 2002)^36^, accumulate in fish tissues^37–39^, transfer up the food chain^39^, and linger in the environment for up to a year post-bloom^37,39^. If a cessation of sound occurred without mortality and/or emigration, fish abundance would be expected to follow the normal seasonal patterns seen in non-bloom years in Sarasota Bay, where fish abundance increases significantly from Jun/Jul to Aug/Sept (E. McCabe, personal communication, 30 June, 2020). Normal non-bloom soniferous fish abundance increases as well, but not significantly (E. McCabe, personal communication, 30 June 2020). In this study, fish sampling stations in proximity to the PALS experienced significant decreases in non-clupeid and soniferous fish CPUEs during bloom conditions (Aug/Sept/Oct) compared to pre-bloom conditions (Jun/Jul).

It is often difficult to identify the specific cause of declines in fish abundance due to the many factors that affect natural mortality, immigration, emigration, prey availability, and habitat use of fishes, however a combination of significant decreases in fish abundance, including species-specific abundances, and shifts in community structure can lend insight into how red tides affect ecological patterns^14^. Gannon et al. (2009) used a weight of evidence approach to show consistent patterns of decreases in fish abundance, species density, and shifts in community structure across five different habitats in Sarasota Bay, Florida during a severe red tide in 2005 and 2006. *K. brevis* red tides were shown to be the major causative environmental factor contributing to these patterns. Similarly, Flagherty et al. (2011) found significant changes in community structure and declines in the annual recruitment of three important recreational sportfish species during this same period in Tampa Bay, Florida, an estuary just north of Sarasota Bay. Species-specific subadult and adult abundances remained consistent with previous years, which the authors hypothesized could be due to differential physiological tolerances to brevetoxin, ontogenetic changes in habitat use, or fish movement into freshwater areas unaffected by the red tide. Similar to the current study, the authors did not find evidence of anoxic conditions within their study area, but the influence of salinity was unclear, as regional and seasonal trends in recruitment and distribution were influenced by variable freshwater-input effects. Walters et al. (2013) used passive acoustic surveys to document a cessation of sand seatrout aggregation sounds coinciding with red tide conditions at ichthyotoxic levels in three sections of Tampa Bay from 2004 to 2005^32^. Acoustic data, along with migration limitations and a subsequent 4-year depression in juvenile sand seatrout abundance, indicated *K. brevis* toxicity as the likely cause of adult mortality and a reduction in spawning. In this study, the combination of decreases in mean sound spectrum levels across all frequency bands during the day and night, decreases in CPUE and SDPUE for non-clupeid and soniferous fish species, and the sudden presence and increase in *K. brevis* concentrations suggests the reduction in both anthropogenic (likely related to decreases in boat density during red tide conditions) and biological sound were due to red tide moving into the study region. Salinity and temperature were significantly higher during pre-bloom conditions compared to bloom conditions, however changes of < 1 °C and < 3 ppt in mean temperature and salinity were unlikely to be biologically meaningful as all species included in these analyses exhibit relatively broad regional distributions and naturally experience a range of seasonal temperatures and salinities (e.g., Sarasota Bay [Manatee County portion, June–October 2017] water temperature 10th and 90th percentiles were 26.6 and 32.0 °C and salinity 10th and 90th percentiles were 30.3 and 34.4 ppt)^40^. Conversely, the mean *K. brevis* concentration in pre-bloom conditions was < 5,000 cells/L while the mean *K. brevis* concentration in bloom conditions exceeded ichthyotoxic levels. Increasing *K. brevis* density was associated with decreasing CPUEs of non-clupeid species, including all species of soniferous fish. These results suggest that the presence of *K. brevis* or their associated brevetoxins directly or indirectly contributed to the changes in the fish community at fish sampling stations near the PALS. Combining results from passive acoustic monitoring and fish sampling analyses suggests that (1) the decrease in mean spectrum sound level likely reflects decreased soniferous fish abundance and species density, and (2) soniferous fish may be a useful indicator of fish abundance and species density for non-soniferous species (excluding clupeids).

Dolphins and manatees also produce sound in the frequency bands analyzed (i.e., 2–20 kHz)^41,42^. Beginning in July 2018, elevated dolphin mortalities were reported along the southwest coast of Florida, and to a lesser extent, involving Sarasota Bay resident dolphins that use the Gulf and bay waters resulting in the declaration of an unusual mortality event (UME) by NOAA along the southwest coast of Florida (N = 196 stranded dolphins as of 22 November 2019)^43^. Seven dolphin carcasses were recovered in Manatee County between June 2018 and January 2019, the same county with the PALS (Mote Marine Laboratory Stranding Investigations Program, unpublished data, 27 November 2019). Not all of these mortalities could be directly attributed to red tide (liver, kidney, feces, and/or stomach contents must have brevetoxin concentrations > 200 ng/g), but many tested positive for brevetoxin^43^. Additionally, 174 Florida manatee mortalities were associated with *K. brevis* between July 2018 and January 2019, with 7 being recovered from Manatee County whose deaths were classified as resulting from red tide and another 6 with red tide suspected to be the cause of death (e.g., animals tested positive for brevetoxin, but had necropsy findings consistent with multiple causes of death; body cavity was exposed to the environment and may have been contaminated; or the carcass could not be recovered for a full necropsy)^44^. The large-scale mortality of sound-producing fish and mammals during the HAB event likely explains at least part of the decrease in sound levels observed.

Establishing a long-term passive acoustic network in advance of HAB events provides baseline information for comparison during and after HABs in addition to identifying critical areas to protect. Baseline data allow researchers to potentially detect shifts in habitat use, acoustic behavior, abundance, and spawning success. For example, distribution and use of spotted seatrout (*Cynoscion nebulosus*) spawning areas have previously been identified via acoustic monitoring^24^ and the sound level during spotted seatrout spawning was found to correlate with the number of eggs produced^45^. Extending acoustic monitoring over multiple years would allow for detection of changes in spawning patterns during and after a HAB. Deviations in spawning patterns and success are particularly important metrics for determining the impact of, and recovery potential from, HABs and other disturbances. The two PALS examined in this study were pilot stations deployed in this region as part of an initiative to build a network of PALS in the Sarasota Bay region for monitoring the region soundscape over time. As part of this monitoring, they collected 67 days of acoustic data before and during a HAB with concentrations at known ichthyotoxic levels. The further development of this network will allow for additional baseline and recovery data to be obtained when future HABs enter the region.

A limitation of using mean spectrum sound level as an indicator of biological change is no species-specific information. Further information can be gained by identifying the species producing specific sounds and examining changes at the species level, however species identification of all sounds produced is a time- and labor-intensive endeavor. While PALS store raw recordings that can be used for in-depth examination of local soundscapes when warranted, the goal of this paper is to show how automated PALS can capture the effects of episodic events and are scalable to cover large spatial areas. A representative soundscape depicted in Fig. 4A of a pre-bloom sound sample illustrates the complexity of overlapping biological signals. However, a comparison between the pre-bloom (Fig. 4A) and during bloom (Fig. 4B) spectrograms shows a clear difference in biological activity that is captured by mean sound spectrum levels across frequency bands. Only changes in soniferous species are detected by PAM; however, sound producing species occur across trophic levels and can serve as indicators for non-soniferous species in a shared niche. Species-level resolution would further elucidate ecological dynamics in a region and inter-trophic interactions during recovery. A better understanding of ecological dynamics and timing of recovery from HABs can be used to bolster recovery from future HAB events and aid commercial and recreational ventures. While identifying the source species for sounds produced provides valuable information, an advantage of using mean spectrum sound levels is that they are comparatively quicker and easier to determine and they summarize the current soundscape in a straightforward manner.

The biological impacts of HABs can persist long after a bloom has dissipated, and well beyond the time fish carcasses stop appearing on beaches. The brevetoxins *K. brevis* produce linger after the bloom and remain in the food web^37–39^. Accordingly, fish abundance, diversity, and community structure take time to recover^5,14^. Prolonged recovery of fish species can negatively impact higher-trophic species such as bottlenose dolphins. Understanding the timing of fish community recovery is important biologically and economically to an affected area. Such insights can help predict recovery time windows of future *K. brevis* blooms. Acoustic monitoring can inform how populations of soniferous species are recovering. Frequency content of fish sounds can relate to body size, therefore informing age structure of sound-producing fish^46^. Additionally, fish species may repopulate the area through mating and/or by moving in from neighboring unaffected areas. Using a passive acoustic network, soniferous fish species movement can be spatially compared to HAB concentration and identify if there were less-affected areas that may have served as sanctuaries for part of the fish population. The current study employed a variety of water sampling schemes (i.e., fixed and opportunistic stations) with an uncommonly high spatial density of *K. brevis* sampling in a partially enclosed water body (Anna Maria Sound, Palma Sola Bay, and Sarasota Bay). This revealed *K. brevis* concentrations that varied over time and space within a bloom. It should be noted that while blooms are typically characterized by cell density, this reflects the presence of unreleased neurotoxins rather than the presence of brevetoxins in the water. The dynamic nature of blooms suggests future studies should be mindful of spatial and temporal resolution when sampling. Areas between *K. brevis* patches, or in lower salinity waters nearby, may serve a vital role in repopulation of fish species throughout the region. Identifying fish sanctuaries during HABs and their spawning areas after a HAB could allow for targeted efforts to bolster and expedite recovery of fish populations.

While the data set presented here is limited to time just a few months before and during the HAB event that entered the region, preliminary observations of data recorded since this HAB event from these and additional stations suggests that sound levels are returning to pre-August 2018 levels. The decrease in mean sound spectrum levels that we documented in 2018 is coincident with decreased fish abundance, decreased fish species density, and increased observations of *K. brevis* in the region, and thus highlights the power of PALS for detecting and monitoring the biological impacts of HAB events. Compared to traditional monitoring methods, PALS can provide real time, non-invasive, monitoring of the impacts of the HABs with higher temporal resolution and comparatively inexpensive implementation, relative to monitoring activities such as seining or trawling for fish assessment. In addition, the ability of PALS to record raw data can be used as a comparison to ground truth mean spectrum sound levels. Real time PAM systems such as PALS can provide the first indication of a biological change and is bolstered by combining multiple ecological survey and sampling methods. In addition, employing a network of PALS can increase spatial resolution and even exceed resolution obtained from traditional sampling methods. Additional benefits of the PALS include the solar-powered feature that lends itself to deployment in areas without power availability while supporting continuous data collection for the length of the deployment. Further, given PALS transmit data immediately over a cell phone network, researchers can avoid the negative health risks associated with being exposed to HABs while conducting traditional water sampling or species-specific surveys. Thus, we recommend that PALS and networks of PALS should be considered for implementation in regions susceptible to environmental disturbance, particularly those known to be home to soniferous species and vulnerable to acoustic disturbance. Additionally, it may be possible to use PALS networks to monitor anthropogenic noise (e.g., boat noise) as an index of human activity.

## Methods

### Karenia brevis data collection

In 2018, a *K. brevis* red tide HAB entered the Sarasota Bay region. Water samples collected as part of separate, ongoing red-tide monitoring programs (Chicago Zoological Society’s Sarasota Dolphin Research Program [SDRP]) investigating spatial and temporal trends in *K. brevis* dynamics in the greater Sarasota Bay region were used to examine *K. brevis* concentrations in relation to the acoustic data recorded by PALS located in the study region. Surface water samples were collected twice per month at five fixed stations from July 2018 onward, throughout the study period at fish sampling sites (described below) located throughout the study region, and opportunistically at bottlenose dolphin sighting locations from September 2018 onward^47^. Each sample was collected in a 20 mL scintillation vial, Utermöhl solution was added for preservation, and the sample was stored at room temperature in darkness until processing. Standard processing involved gentle agitation of the vial to resuspend the cells, taking a 1 mL subsample, and counting all *K. brevis* cells. High cell density samples underwent 1:10 serial dilution with filtered water of matching salinity. All counts were converted to cells/L for reporting. All sample collection and processing used standard protocols^48–50^ and are explained in more detail in Gannon et al. (2009).

### Acoustic data collection

Two land-based marine PALS (Fig. 1) were deployed in Sarasota Bay, Florida, as part of an initiative developed to establish a network of PALS in Sarasota Bay to better understand the biology of the region’s marine life and how it responds to disturbances, such as red tide and boat noise. One station was deployed on Longboat Key along the Intracoastal Waterway in northern Sarasota Bay (LBK) on 16 June 2017, while the other was deployed near the mouth of Palma Sola Bay (PSB) on 02 November 2017 (Fig. 3). Additional PALS have since been deployed in this region but are not analyzed here as they were not active during the HAB event.

PALS are open-source, solar-powered PAMS designed by Loggerhead Instruments, Inc. that continuously record and collect acoustic data from the marine environment via land-based systems wired to submerged hydrophones (HTI-96-min; sensitivity -180 dBV/uPa; High-Tech Inc.) (https://github.com/loggerhead-instruments/PALS). Hydrophone signals are digitized by a recording board with 16-bit resolution. The incoming acoustic data stream is processed in real-time with a Hanning window followed by a 256-point fast Fourier transform (FFT). Mean sound spectrum levels in user-defined frequency bins are averaged over 5-min intervals, and then reported over a cell phone network (Particle Electron) to a cloud database running on Amazon Web Service (DynamoDB) and website where they are immediately available. All raw acoustic data are stored on microSD cards located within the PALS (Fig. 1). Each file is time-stamped with UTC time (derived from cell network) and named according to the serial number of the board and the date and time of the recording. PALS can generate 3 TB of raw acoustic data (.wav files) per year while running continuously with a 44.1 kHz sample rate.

The PSB and LBK PALS were programmed to calculate and report mean spectrum levels (dB re 1 μPa^2^/Hz) for each 5 min interval in the following frequency bands: 0.172–1 kHz, 1–2 kHz, 2–5 kHz, and 5–20 kHz. These bands were selected to characterize both anthropogenic sounds, such as vessel noise that is typically concentrated in lower frequency bands (e.g., < 5 kHz), and biological sounds, such as those produced by fish (typically within 0.5–5 kHz)^23,28,45,51^, manatees (typically within 2–20 kHz)^42^, and bottlenose dolphins (whistles are typically within 2–20 kHz, and portions of echolocation clicks and burst pulses fall within this range as well)^41^. Hydrophones were calibrated before deployment and there was no evidence of sensitivity loss during deployment or upon retrieval.

### Fish and environmental sampling methods

Fish data were obtained from a separate ongoing effort designed to characterize long-term (since 2004) seasonal trends in fish populations in the greater Sarasota Bay region (SDRP). Fish were collected from survey sites that were randomly selected amongst seagrass beds, using a purse-seine net (183 × 6.6 m, 2.5 cm stretch mesh). Fish were identified to species, measured, counted, and released. Fish counts and species data were translated into relative abundance expressed as CPUE and SDPUE, with each deployment (set) of the seine net being the unit of effort. Gannon et al. (2009) found that clupeids have a different relationship with red tide than other fish species. During a previous red tide event in the same region, clupeids were positively associated with *K. brevis* concentration while non-clupeid CPUE and SDPUE showed a negative relationship with *K. brevis* concentration. Changes in CPUE and SDPUE were considered separately for non-clupeid fish species and for soniferous, or sound-producing, fish species (soniferous fish is a subset of non-clupeid fish) (Table 3). Fish species were classified as soniferous if they are known to produce sound under “normal” circumstances (i.e., not being electrically stimulated) in Fish and Mowbray (1970), Breder (1968), or based on auditory observations by the researchers during fish surveys^52,53^. Bottom temperature, DO, and salinity were measured approximately 15 cm above the substrate at each station with a YSI Pro2030 multiprobe and were recorded along with measurement depth. See Gannon et al. (2009) for more detail regarding fish sampling methods and procedures. Fish sampling research was conducted in accordance with the relevant guidelines and regulations. It was authorized by the Florida Fish and Wildlife Conservation Commission (Special Activity License number 16-0809-SR) and Mote Marine Laboratory’s Institutional Animal Care and Use Committee (17-10-RW2).

### Data analysis

Acoustic data were processed and compared to data regarding *K. brevis* concentrations, environmental parameters, non-clupeid fish CPUE/SDPUE and soniferous fish CPUE/SDPUE using custom written code in R v3.5.2^49^. Only data recorded from 1 June 2018 to 13 October 2018 were included in our analyses because they represented equal number of days (*n* = 67) before and after *K. brevis* cells were first observed in the study region at or greater than ichthyotoxic levels (see “Results”), and for which we generally had continuous acoustic data (i.e., few technical issues with the PALS). Reported mean sound spectrum levels for each frequency band calculated every five minutes were converted into sound pressure (µPa) to complete statistical tests. Results were converted back to spectrum levels (dB re 1 μPa^2^/Hz) for reporting. Diurnal patterns in mean sound spectrum levels were calculated using daytime and nighttime periods, which were determined using the midpoint sunrise and sunset times obtained from the NOAA Solar Calculator^54^ for the midpoint location of our study site (Latitude N 27.44762, Longitude W − 82.67351) on 1 June 2018 and 13 October 2018: daytime = 0703–1943 EDT and nighttime = 1944–0702 EDT). All times are reported in local (EDT) time (UTC − 4 h).

*K. brevi*s, fish, and environmental data were restricted to those samples collected for other SDRP research projects during 1 June 2018 to 13 October 2018 within 4 km north and west of the PSB PALS, and 4 km south and east of the LBK PALS. Data analyses and visualizations were made in R v3.5.2 using the following packages: *cowplot*^55^, *lubridate*^56^, *rnaturalearth*^57^, *sf*^58^, *tidyverse*^59^, and *viridis*^60^. Figure 3 was made using the Florida Shoreline (1 to 40,000 Scale) shapefile available from the Florida Fish and Wildlife Conservation Commission GIS & Mapping Data Downloads (https://geodata.myfwc.com/)^61^. All statistical analyses were performed using the *stats* package^62^, and assumed a significance level of 0.05. Values are presented as mean [median, range], unless otherwise specified. The Wilcoxon Rank Sums test, a non-parametric test, was used to compare means because sample sizes were small and the data were not normally distributed^63–65^.

## Data Availability

All data are available upon reasonable request to the corresponding author.
